# Tetrahydrobiopterin responsiveness in a series of 53 cases of phenylketonuria and hyperphenylalaninemia in Iran

**DOI:** 10.1016/j.ymgmr.2015.01.001

**Published:** 2015-01-28

**Authors:** Aria Setoodeh, Bahram Yarali, Ali Rabbani, Shohreh Khatami, Sedigheh Shams

**Affiliations:** a Division of Pediatric Endocrinology, Children's Medical Center, Tehran, Iran; b Division of Pediatric Neurology, Children's Medical Center, Tehran, Iran; c Growth and Development Center, Children's Medical Center, Tehran, Iran; d Division of Biochemistry, Pasteur Institute, Tehran, Iran; e Division of Pathology, Children's Medical Center, Tehran, Iran

**Keywords:** Phenylketonuria, Hyperphenylalaninemia, Sapropterin, Tetrahydrobiopterin, Kuvan®

## Abstract

To determine the prevalence of 6R-Tetrahydrobiopterin (BH4) responsive phenylketonuria (PKU) in 53 cases of patients with various classification of hyperphenylalaninemia and PKU Excluding the BH4 deficient type referring to children's medical center in Iran (phenylalanine 360–2420 μmol/L), the single dose of 20 mg/kg (Kuvan®) and duration of 24 h was used.

**Results:**

Among the 4 different categories of mild hyperphenylalaninemia requiring treatment, mild, moderate and classic PKU, the BH4 responders were 90%, 35.7%, 5.6% and 0% respectively after 24 h.

**Conclusion:**

BH4 responsiveness is more prevalent in mild hyperphenylalaninemia and mild PKU patients in Iran.

## 1 Introduction

Phenylketonuria (PKU) (OMIM 261600) is an inborn error of phenylalanine (PA) metabolism with severe mental retardation and microcephaly if left untreated. The mainstay of treatment has been restriction of PA in diet since more than 40 years ago, which prevents the severe intellectual impairment. But with the odd taste and odor of this artificial diet, the adherence to this diet for life has been very challenging which results in a high rate of treatment non-compliance and failure which is increased with age [1], [2]. A variety of adverse neurocognitive and psychiatric out comes, such as deficit in executive functions, and psychiatric symptoms such as anxiety, depression and phobias can develop later in life after relaxation of PA control [3], [4], [5], [6], [7], [8].

Therefore there has always been an urge to find some other therapies besides the diet to increase the PA tolerance or even replace the diet. In this respect large neutral amino acid (LNAA) supplementation is one of the options. LNAA have been proposed as a therapy for PAH deficiency based on their ability to block uptake of PA from the intestine and at the blood–brain barrier. A single clinical trial demonstrated reduction of blood PA by 40% after a standard low PA medical food supplemented with LNAA at dose of 0.5 or 1 g/kg of body weight [9]. Treatment is not an alternative to low PA dietary treatment in children and currently there is very limited experience in adult patients and should be avoided in pregnant women.

Another compound which entered phase III clinical trial in 2013 is polyethylene glycol–conjugated phenylalanine ammonia lyase (PEG–PAL), that appears to lower the blood PA levels, by a mechanism independent of PAH. The only drawback is the daily subcutaneous injections.

Phenylalanine hydroxylase (PAH) enzyme cofactor, tetrahydrobiopterin (BH4) reduces plasma PA level in some PKU patients, hence the responsiveness must be determined with a BH4 loading test. The arbitrary responsiveness definition is > 30% reduction in blood PA and 20–29% is defined as partial responsiveness. There are different protocols from 24 h to several weeks all around the world.

## 2 Method and material

### 2.1 Patient

In order to assess the BH4 responsiveness in Iranian patients with PKU and hyperphenylalaninemia, the 24 h protocol with (Kuvan®), single dose 20 mg/kg was done. A total of 53 (28 males and 25 females) cases of hyperphenylalaninemia and PKU patients more than 4 years of age with the age range of 4–24 years referring to the children's medical center were divided in to 4 different groups based on the classification by national Institute of health PKU conference (http://www.team-share.net/phenylketonuria_Scientific_Review_Conference/Overview.aspx).

The study was done between 2012 and 2013. 10/53 (18.8%) of the patients with age range (5.2 ± 1.27) had mild hyperphenylalaninemia requiring treatment (blood PA 360–< 600 μmol/L), 14/53 (26.4%) with age range of (7.66 ± 2.88) had mild PKU (600–< 900 μmol/L), 18/53 (34%) with the age range of (11.45 ± 5.5) had moderate PKU (900–< 1200 μmol/L), and 11/53 (20.8%) with the age range of (11.37 ± 6.23) had classic PKU (> 1200 μmol/L).

The frequency of different categories is shown in Table 1, the age range and the mean ± SD is shown in Table 2.

**Table 1:** Frequency of patients in different categories.

PA levels	Frequency	Percent
360–< 600	10	18.8
600–< 900	14	26.4
900–< 1200	18	34.0
> 1200	11	20.8
Total	53	100.0

**Table 2:** Age range in different categories.

Age (years)					
PA levels	N	Minimum	Maximum	Mean	SD
360–< 600	10	4	7.5	5.2	1.27
600–< 900	14	4.16	14	7.66	2.88
900–< 1200	18	4.25	22.4	11.45	5.5
> 1200	11	4	24	11.37	6.23

### 2.2 BH4 loading test

All the patients had been examined for neopterin and biopterin of urine and DHPR activity on blood spots in order to exclude BH4 deficiency before the test. Also they were given partial diet liberalization such that at the morning of the test the PA level would be more than 400 μmol/L. Then Kuvan® was given at a dose of 20 mg/kg and PA levels were checked by HPLC method at times 0–4–8 and 24 h later and a positive response was defined as a reduction of more than 30% in the blood PA level.

## 3 Results

The test was done on 53 cases of PKU more than 4 years of age in the children medical center.

**Fig. 1:**
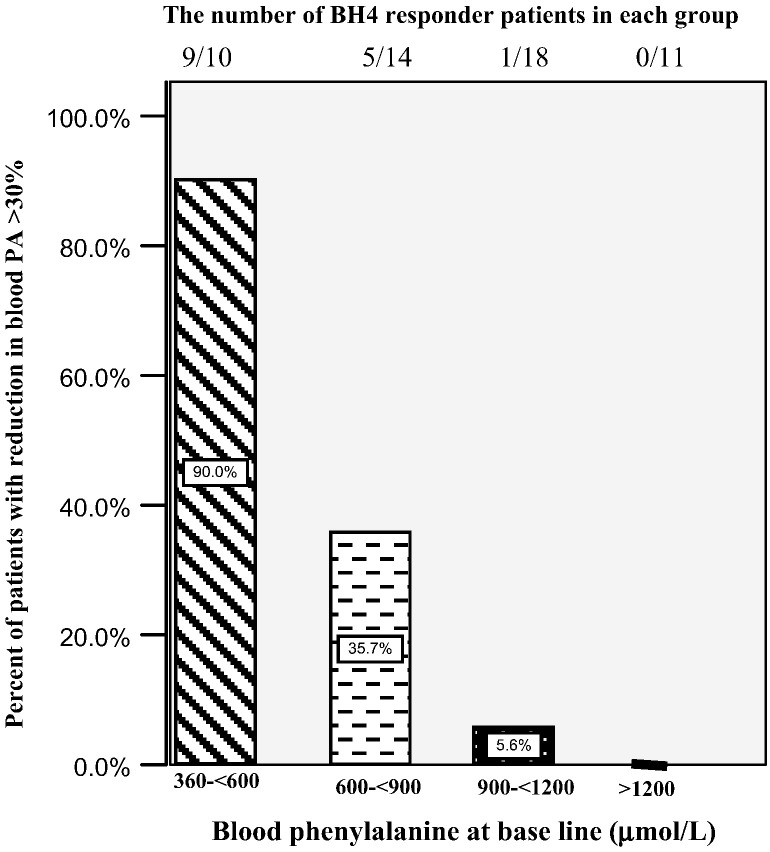
The number of BH4 responder patients in each group.

The responses in different categories are shown in Fig. 1. 90% (9/10) of patients with mild HPA and 35.7% (5/14) of mild PKU and 5.6% (1/18) of moderate PKUs had the positive response (P < 0.0001), none of the severe PKU patients responded to the test. Totally among the 53 cases, 28.3% of patients responded to the test.

## 4 Discussion

PKU has been diagnosed through new born screening (NBS) program in the United States of America and many European countries since 1960. NBS for classical PKU has been established in Iran since the years 2007 and extended for non-classical PKU since 2010. The incidence has been reported by ministry of health to be 1 in 5000 live births.

Access to low protein foods is a big challenge in Iran as they are more costly than the non-modified counterparts and this cost is not covered by the insurance companies, therefore the patients may take benefit of other medication besides regimen. We did this survey on 53 patients between 2012 and 2013 and to our best of knowledge, this is the first report of BH 4 responsiveness in Iran.

Sapropterin dihydrochloride is the biologically synthetic active formulation of BH4, and Kuvan® (Merck Serono, Geneva) is the only commercial formulation of Sapropterin approved by FDA in 2007 and by EMEA in 2008. It increases residual activity of PAH mostly by a pharmacological chaperone action [10], even younger than 4 years of age [11].

Though BH4 loading test was initially used for discrimination between PKU and BH4 deficiency, but it has been a useful tool for the selection of BH4 responsive cases of PKU since Kure et al. showed that four of five patients with HPA responded to oral BH4 therapy by lowering the PA levels [10]. Since then many researches were done in different countries and showed that 10–60% of patients with PKU may be BH4 responsive depending on the mutation prevalent in different population [12].

Also efficacy of BH4 has been evaluated in increasing PA tolerance in children with classical PKU [12], [13], [14], [15], [16]. Most Sapropterin responsive patients have a rapid decline in blood PA level, but occasionally a delay of 2–4 weeks is seen.

Another challenging issue in PKU is maternal PKU in which the maintenance of blood PA levels between 120 and 360 μmol/l prior to conception and maintaining them throughout gestation is mandatory in order to prevent the teratogenic effects of high PA levels. Sapropterin responsiveness should be determined before pregnancy in this group. Sapropterin therapy can be a useful tool for the responsive patients if the safe PA levels cannot be achieved by diet therapy and in noncompliant patient [17]. Furthermore the use of BH4 during pregnancy in BH4 responsive pregnant women is a very good option to reach the strict blood PA target levels. In an European experience in 8 pregnant cases (4 mild HPA, 3 mild PKU and 1 classic PKU) treated with sapropterin, all the off springs but one were delivered normally with normal weight, length and head circumference. The only complication in an offspring was in a mother who had classic PKU (PA > 1200 μmol/L) in the first trimester who carried two nonresponsive mutations and sapropterin was added to diet to optimize and accelerate metabolic control while she wanted to pursue her pregnancy. The off spring died with potter syndrome related to lack of amniotic fluid mostly related to absence of metabolic control in the first trimester of pregnancy. This experience though shows that sapropterin treatment may result in a better metabolic control and increase in protein intake and result in normal anthropometry of non-PKU off spring, but it should be restricted to those women to be clear responder to BH4 prior to pregnancy [18].

An expert panel, searching through PubMed, published the results of 194 papers in English till May 2012. Overall 37% of patients with HPA were responsive to BH4. They reported that there is considerable heterogeneity in response to BH4 loading among mutations and even for the same mutant alleles in siblings considering the existence of modifying factors.

Genotype maybe predictive of Sapropterin response, but genotype–phenotype correlations are imperfect.

In a diet for life treatment many issues such as compliance, palatability, growth, wellbeing and quality of life (Qol) should be undertaken.

In a systemic review of literature in PubMed, Scopus and Psych Info performed in order to assess the outcome data over the last decade in diet-alone early-treated PKU patients, revealed that there is a growing body of evidence suggesting that neurocognitive, psychological, Qol, growth, nutrition and bone parameters are suboptimal [19].

Bone formation and resorption markers have been found to be significantly reduced in diet treated PKU children to healthy controls, and osteopenia and osteoporosis has been detected in adult PKU patients [21]. These may be explained by dietary deficiency in protein, calcium, vitamin D or trace elements. In a 28 series of phenylketonuric patients, mostly 78.6% with a good dietary compliance with an age range of 10–33 years bone density at the lumbar spine by (Dual-Energy X-Ray Absorptiometry) showed that 50% of patients had a defect in bone mineralization that could not be attributed to vitamin D concentration [20].

In a long term follow-up and outcome of 94 patients with low PA-diet treatment alone and 53 patients treated with combination of low PA diet and sapropterin, the investigators reported an improvement in Qol for 49.6% of patients, also an improvement in compliance with diet in 47% of patients in latter than the former group [21].

Because of the poor compliance with the low PA diet after early childhood, new approaches are needed to reduce PA concentration in plasma and brain with potential to liberalize the diet by allowing for a greater daily PA allowance.

## 5 Conclusion

With the biggest challenge to living with PKU being the lifelong adherence to very restricted diet, the results of this survey suggest that sapropterin treatment may allow a subset of subjects mostly mild HPA and mild PKU and some cases of moderate PKUs to reduce the need for PA-free protein diet and still achieve the standard goals of treatment.

## Conflict of interest

The authors declare no conflict of interest.
